# *LDLR* gene rearrangements in Czech FH patients likely arise from one mutational event

**DOI:** 10.1186/s12944-024-02013-3

**Published:** 2024-02-02

**Authors:** Kateřina Konečná, Petra Zapletalová, Tomáš Freiberger, Lukáš Tichý

**Affiliations:** 1 Centre of Molecular Biology and Genetics, University Hospital Brno, Jihlavská 20, Brno, 625 00 Czech Republic; 2 National Centre for Biomolecular Research, Faculty of Science, Masaryk University, Kamenice 5, Brno, 625 00 Czech Republic; 3 Faculty of Medicine, Masaryk University, Kamenice 5, 625 00 Brno, Czech Republic; 4 Centre for Cardiovascular Surgery and Transplantation, Pekařská 53, 656 91 Brno, Czech Republic

**Keywords:** Low-density lipoprotein receptor, LDLR, Familial hypercholesterolemia, Alu, Rearrangements, Breakpoints

## Abstract

**Background:**

Large deletions and duplications within the low-density lipoprotein receptor (*LDLR)* gene make up approximately 10% of *LDLR* pathogenic variants found in Czech patients with familial hypercholesterolemia. The goal of this study was to test the hypothesis that all probands with each rearrangement share identical breakpoints inherited from a common ancestor and to determine the role of Alu repetitive elements in the generation of these rearrangements.

**Methods:**

The breakpoint sequence was determined by PCR amplification and Sanger sequencing. To confirm the breakpoint position, an NGS analysis was performed. Haplotype analysis of common *LDLR* variants was performed using PCR and Sanger sequencing.

**Results:**

The breakpoints of 8 rearrangements within the *LDLR* gene were analysed, including the four most common *LDLR* rearrangements in the Czech population (number of probands ranging from 8 to 28), and four less common rearrangements (1–4 probands). Probands with a specific rearrangement shared identical breakpoint positions and haplotypes associated with the rearrangement, suggesting a shared origin from a common ancestor. All breakpoints except for one were located inside an Alu element. In 6 out of 8 breakpoints, there was high homology (≥ 70%) between the two Alu repeats in which the break occurred.

**Conclusions:**

The most common rearrangements of the *LDLR* gene in the Czech population likely arose from one mutational event. Alu elements likely played a role in the generation of the majority of rearrangements inside the *LDLR* gene.

**Supplementary Information:**

The online version contains supplementary material available at 10.1186/s12944-024-02013-3.

## Background

Familial hypercholesterolemia (FH) is one of the most common autosomal dominant disorders. The worldwide prevalence of FH in the general population is around 1:300 for heterozygous FH and around 1:400,000 for homozygous FH [1, 2]. FH is characterized by elevated levels of low-density lipoprotein (LDL) cholesterol, leading to a higher risk of cardiovascular disease [3]. Untreated males with clinically diagnosed heterozygous FH have a 50% risk of myocardial infarction by 50 years of age, whereas untreated females have a 12% chance by age 50 and a 30% chance by age 60 [4, 5].

FH can be caused by either a single variant (heterozygous FH) or two variants (homozygous FH) in FH-associated genes, with homozygous FH having a more severe phenotype [6]. The three genes most commonly associated with FH are the LDL receptor (*LDLR*), apolipoprotein B (*APOB*) or proprotein convertase subtilisin/kexin type 9 (*PCSK9*) (reviewed in [7]). During genetic testing of Czech patients with a clinical diagnosis of FH, a pathogenic variant in the *LDLR* gene was found in 22% of patients, while 11% of patients carried an *APOB* variant, and the rest remained without a known causal variant [8].

The *LDLR* gene was the first gene associated with FH, and its variants remain the most common cause of FH [8–10]. The *LDLR* gene is located on chromosome 19, spans 45 kbps and contains 18 exons. It encodes a protein that is 860 amino acids long, with the first 21 amino acids making up the signal sequence that is cleaved off during processing, giving rise to a mature protein of 839 amino acids [11]. The *LDLR* gene contains 98 Alu repeats, out of which 3 are located in the 3’UTR, while the rest reside inside introns [12].

Large rearrangements of the *LDLR* gene are a relatively common cause of FH [8], possibly due to Alu-mediated rearrangements caused by the high density of Alu elements inside *LDLR* introns.

Alu elements are the most common sequence elements in the human genome, numbering over one million copies and making up 11% of the human genome [13]. Alu elements are transposable elements belonging to the class of short interspersed nuclear elements (SINE). The name Alu is based on the presence of an *AluI* restriction site within these elements [14]. The elements were originally derived from 7SL RNA [15].

**Fig. 1:**
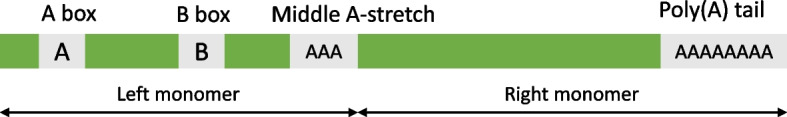
The structure of an Alu element. One Alu element is about 300 bps long and consists of two monomers separated by a short A-rich sequence. The left monomer contains a bipartite RNA polymerase III promoter, composed of an A and a B box. The right monomer contains an insertion, rendering it 31 bps longer than the left monomer, and ends with a poly-A tail [16]

The typical structure of an Alu element is shown in Fig. 1. Alu elements can be divided into three major subfamilies: the oldest AluJ, younger AluS and the youngest AluY. The subfamilies can be distinguished based on the presence of diagnostic variants. Individual Alu elements may differ from each other both by subfamily-specific variants (which are shared by all Alu elements belonging to a specific subfamily due to common origin from the same ancestral Alu) and by random variants, which are specific to the individual element (since these variants started accumulating after the individual Alu element had integrated into the genome). Youngest Alus have the largest number of subfamily-specific variants and the lowest number of random variants (reviewed in [16]).

Alu elements have been implicated in the generation of genomic rearrangements [11, 17, 18]. Recombination between two Alus can generate a new chimeric Alu element, with the breakpoint located within a microhomology region common to both Alus. In this publication, the word „breakpoint “ is used to describe the region of microhomology in which the break occurred because the exact location of the break within this microhomology region cannot be determined. Alu-mediated rearrangements were previously thought to arise mainly through non-allelic homologous recombination (NAHR) or non-homologous end joining (NHEJ) [19]. Recently, other mechanisms have been suggested for the generation of Alu-mediated rearrangements, such as microhomology-mediated end joining (MMEJ), fork stalling and template switching/microhomology-mediated break-induced replication (FoSTeS/MMBIR), single-strand annealing (SSA), and others [20–22].

In the present study, we characterized the breakpoints of eight rearrangements of the *LDLR* gene found in the Czech population, in some cases correcting the breakpoints published in a previous study [23]. Additionally, we characterized the breakpoints of all probands carrying the same rearrangement (duplication or deletion of the same exons as characterized by multiplex ligation-dependent probe amplification (MLPA)) to explore the hypothesis that all Czech probands with each rearrangement share the same breakpoint inherited from a common ancestor.

## Methods

### Patients

Patients have been identified through the Czech MedPed project (Make Early Diagnosis to Prevent Early Deaths), which aims to identify and treat patients with FH in the Czech Republic [24]. As of 4th March 2022, there have been 8,918 patients with FH (6,753 unrelated families) in the Czech MedPed datase.

Patients included in this study carried a large rearrangement in the *LDLR* gene, which had been previously determined by multiplex ligation-dependent probe amplification (MLPA) analysis as part of their diagnosis [8, 23, 25]. Large deletions and duplications make up approximately 10% of *LDLR* pathogenic variants found in Czech patients [8]. Some of these large rearrangements number among the most common pathogenic variants found in Czech FH patients, such as deletion of exons 9–14 or duplication of exons 2–6, which have been found in 28 and 26 independent families, respectively, making them the 6th and 7th most common pathogenic variants in Czech FH patients.

For each studied rearrangement, all known Czech families carrying a specific rearrangement were analysed, apart from one proband with duplication of exons 2–6, whose DNA was not available for analysis. The numbers of analysed families can be found in Table 1. Promoter_exon2del and exon3_12del were not included in the common ancestry analysis because these rearrangements have only one known proband.

**Table 1:** Number of analysed probands and frequencies of studied rearrangements in the Czech population

Rearrangement	Number of known families in CR	Number of analysed families	Frequency (%) ^a^
promoter_exon2del	1	1	0.09
exon2_6dup	26	25	2.41
exon3_12del	1	1	0.09
exon4_8dup	2	2	0.19
exon5_10del	4	4	0.37
exon9_14del	28	28	2.59
exon9_15del	11	11	1.02
exon16_18dup	8	8	0.74

*CR* – the Czech Republic,

^**a**^Frequency of the rearrangement among other potentially causal *LDLR* variants (pathogenic or variants of unknown significance) found in the Czech population. Frequency was determined as the percentage of probands carrying the specific rearrangement out of all probands with a pathogenic or VUS *LDLR* variant recorded in the Czech MedPed database as of 4th March 2022

### Breakpoint analysis

Breakpoint junction sequences were determined by PCR or nested PCR and subsequent Sanger sequencing. Nested PCR was used in cases where simple PCR did not yield a specific product and it was not possible to design new primers in close proximity of the presumed breakpoint due to the high density of repeats inside *LDLR* introns. Sequences were amplified by Taq polymerase (Thermo Fisher Scientific) using primers indicated in Supplementary Table S1 within Additional file 1, and sequenced using Big Dye Terminator v1.1 Cycle Sequencing Kit (Thermo Fisher Scientific) followed by analysis on 3130xl Genetic Analyzer (Applied Biosystems). To determine the breakpoint location, sequences obtained by Sanger sequencing of the breakpoint region were compared to the reference sequence NG_009060.1 or analysed using the Basic Local Alignment Search Tool (BLAST) of the NCBI [26]. The description of variants is based on the reference sequence NG_009060.1(NM_000527.4). Variants are described in accordance with the recommendations of the Human Genome Variation Society (v20.05).

Primers were designed so that only the allele with a deletion or duplication and not the WT allele gave a product. The specificity of primers was confirmed by including negative control DNA – DNA from patients that had no rearrangements of the *LDLR* gene according to a previous MLPA analysis [8, 23, 25]. Agarose gel electrophoresis of the control PCR products gave no product while the DNA of patients with the duplication gave one specific band on the gel. Primers were initially designed to flank the breakpoints published in a previous study [23]. In some cases, primers identical to those published in a previous study were used [23]. In the case of exon3_12del, the primer design was based on the approximate location of the breakpoint as determined by CNV analysis of NGS data.

### Haplotype analysis

To determine if the rearrangements were inherited from a common ancestor, we aimed to determine the haplotype associated with a specific rearrangement in different families carrying the same rearrangement. Two strategies were used to obtain a haplotype. First, the sequences obtained during sequence analysis of the breakpoints were searched for sequence variants in close proximity of the breakpoint in all families (see Breakpoint analysis). Because the primers were designed to only amplify the allele carrying the rearrangement, all observed variants are *in cis* with the rearrangement.

Secondly, common variants found in the *LDLR* gene were genotyped in probands and their relatives (if available). Sanger sequencing was used to genotype polymorphisms commonly found within or near *LDLR* exons. Variants for genotyping were chosen based on their frequency in the European non-Finnish population according to gnomAD v4.0.0. The population frequency of chosen variants was between 0.063 and 0.75. A list of chosen variants and their population frequencies can be found in Table 2. Primers used for the amplification of genotyped regions can be found in Supplementary Table S2 within Additional file 1.

**Table 2:** Frequent variants used for haplotyping

Variant ^a^	Protein change	Frequency (gnomAD) ^b^	Location
c.81C>T	p.Cys27Cys	0.1232	exon 2
c.190+56G>A	-	0.06341	intron 2
c.1706-55A>C	-	0.5890	intron 11
c.1725C>T	p.Leu575Leu	0.1309	exon 12
c.1773C>T	p.Asn591Asn	0.4461	exon 12
c.2232A>G	p.Arg744Arg	0.7534	exon 15
c.2548-42A>G	-	0.4876	intron 17

A list of all variants used in haplotype analysis. Not all variants were used for haplotyping all rearrangements

^**a**^Description of variants is based on the reference sequence NG_009060.1(NM_000527.4)

^**b**^Population frequency of the variant in the European non-Finnish population according to the gnomAD database v4.0.0

Together, the variants found in close proximity of breakpoints and common variants further away in the *LDLR* gene were used to create a haplotype.

### Analysis of repeats

Repetitive sequences were identified using RepeatMasker version open-4.0.9 [27]. To determine the sequence identity of Alu elements present at both sides of the breakpoint junctions, Emboss NEEDLE was used for pairwise global alignment of the two repeats surrounding the breakpoints [28].

### NGS

To narrow down the breakpoint position, copy number variation (CNV) analysis of next-generation sequencing (NGS) data was performed. The sequencing library was prepared using the method of Hybridization Capture-Based Target Enrichment for NGS (Roche), following KAPA HyperCap Workflow v3.0. Genomic DNA was enzymatically fragmented using the KAPA HyperPlus kit (Roche), ligated to KAPA Universal Adapters (Roche). The sample library was amplified by PCR using KAPA UDI Primer Mixes (Roche) and KAPA Hifi HotStart Ready Mix (Roche), purified using KAPA HyperCapture Bead kit (Roche), and hybridized to KAPA Hyper Choice MAX 3 Mb probes (Roche), PCR amplified and purified using KAPA HyperCapture Bead kit (Roche). Enriched samples were sequenced on the NextSeq sequencer (Illumina).

Data analysis was performed in QIAGEN CLC Genomics Workbench v.21.0.5. CNV regions within NGS data were identified by applying a threshold to adjusted fold-change. The threshold used was < -1.38 fold-change (adjusted) for a deletion and > 1.29 fold-change (adjusted) for a duplication. These in-house thresholds have previously been determined by analysis of control samples by the same method. The control samples were DNA samples in which a deletion or duplication had previously been identified by MLPA analysis. Thresholds were set as the minimum/maximum fold-change (adjusted) identified in a region that has been determined to be deleted/duplicated (respectively) by MLPA.

Analysis in QIAGEN CLC Genomics Workbench only allowed for approximate determination of the breakpoint region. Next, we attempted to identify the exact location of the breakpoint by visually examining the data in Integrative Genomics Viewer (IGV) v.2.5.0 [29].

## Results

Breakpoints of *LDLR* rearrangements in Czech FH patients have already been published in 2010 by Goldmann et al. [23]. The goal of this study was to analyse the breakpoints in all known families with these rearrangements available 10 years later in order to determine whether all Czech families share the same breakpoint. A secondary goal was to analyse the breakpoint region for the presence of Alu repeats to determine the role of these repetitive elements in the generation of rearrangements within the *LDLR* gene.

### Breakpoint characterization

To determine the breakpoint position of selected rearrangements of the *LDLR* gene, PCR or nested PCR and Sanger sequencing were used.

The studied rearrangements included 5 deletions and 3 duplications within the *LDLR* gene. Among these were the four most common *LDLR* rearrangements in the Czech population (number of probands ranging from 8 to 28), and four less common rearrangements (1–4 probands) (Table 1). The size of analysed rearrangements ranged between 6 and 17 kbps. All of the breakpoints occurred within the introns of the *LDLR* gene, except for duplication of exons 16–18, which involved the 3’UTR. The breakpoint position and size of the rearrangement determined in this study is shown in Table 3, while the sequence is shown in Additional file 2. For each of the analysed rearrangements, all Czech probands with a specific rearrangement shared identical breakpoint positions. The breakpoint of an Alu-Alu rearrangement is typically found inside a microhomology region – a region with identical sequence in both Alus surrounding the breakpoint. The exact position of a breakpoint within this microhomology region cannot be determined.

**Table 3:** Position of breakpoints, and their comparison with a previous study

Rearrangement	Breakpoint determined in the current study ^a^	Breakpoint according to a previous study [23]^b^	Difference between the breakpoints determined in each study (bps) ^c^	Duplication/ deletion size (bps) ^d^
promoter_exon2del	c.-1823_190+566del	=		13,186
exon2_6dup	c.67+3545_940+917dup	c. 67+3968_940+296dup	1,044	15,272
exon3_12del	c.191-481_1846-1096del	c.190+984_1846-1160del	790	16,814
exon4_8dup	c.314-443_1187-385dup	c.314-446_1187-386dup	2	8,117
exon5_10del	c.695-67_1586+371del	=		7,636
exon9_14del	c.1186+700_2141-545del	=		10,291
exon9_15del	c.1187-169_2312-790del	=		14,110
exon16_18dup	c.2312-2067_*1216dup	c.2311+1941_*1216dup	656	6,592

Breakpoints of these rearrangements in the Czech population have already been determined in 2010 by Goldmann et al. [23], presumably using the same patients. Surprisingly, the position of some of these breakpoints was determined to be different in the current study (see Additional file 3 for more details)

^a^Description of variants is based on the reference sequence NG_009060.1(NM_000527.4)

^b^" = " denotes that the breakpoint was the same in both studies

^c^The difference was computed as the difference between the size of the deletion/duplication as determined in each study

^d^size of the rearrangement (either duplication or deletion) based on the breakpoints determined in the current study

### Haplotype analysis

To determine if each rearrangement was inherited from a common ancestor in all patients with a specific rearrangement in the Czech population, the haplotypes associated with specific rearrangements were analysed in different families carrying the same rearrangement.

First, the sequences obtained during Sanger sequencing analysis of the breakpoints were searched for sequence variants within a few hundred bps away of the breakpoint. Two rearrangements were found in only one proband, and thus were excluded from the haplotype analysis. Six rearrangements were found in more than one family. In 5 out of these 6 rearrangements, Sanger sequencing revealed at least one sequence variant in close proximity of the breakpoint (Table 4; Additional file 2). Two of these variants were unique and specific to a certain rearrangement, while the others were common in the population. For each of these 5 rearrangements, the variants were identical in all analysed families carrying the same rearrangement.

**Table 4:** List of variants found in close proximity of the breakpoint

Rearrangement	Variant ^a^	Frequency (gnomAD) ^b^	Distance from the breakpoint (bps) ^c^	Length of sequenced region (bps)
exon2_6dup	c.940+793_940 + 795dup	0.5962	100	401
exon4_8dup	none found	-	-	256
exon5_10del	c.694+285A>C	0.4519	609	759
	c.694+341G>A	0.5075	553	
exon9_14del	c.1186+684A>C	NR^d^	11	277
exon9_15del	c.2312-759_2312-754del	0.04835	20	446
	c.1187-269_1187-266TG[4]	NR^d^	64	
exon16_18dup	c.*1216dup	0.7005	1	579

These variants were identical in all families with each breakpoint. Only those rearrangements that were found in more than one family are listed. In both families with duplication of exons 4–8, no variants were found in the sequenced region of 256 bp

^a^Description of variants is based on the reference sequence NG_009060.1(NM_000527.4)

^b^Population frequency of the variant in the European non-Finnish population according to the gnomAD database v4.0.0

^c^Distance of the variant from one end of the microhomology region surrounding the breakpoint

^d^NR – not reported in gnomAD v4.0.0 – signifies a unique variant associated with a specific rearrangement

Notably, all 28 probands with exon9_14del carried a novel, previously unreported variant NG_009060.1(NM_000527.4):c.1186+684A>C, which was situated 11 bps away from the breakpoint (11 bps from the 5’ end of the microhomology region). All probands with exon9_15del carried a rare variant c.1187-269_1187-266TG[4], which has not been reported in gnomAD v4.0.0, but it has been reported as a rare variant in 14KJPN, 8.3KJPN and Korea1K databases in Japanese and Korean population, respectively (see rs1555805065). In four rearrangements (exon2_6dup, exon5_10del, exon9_15del, exon16_18dup), all probands carried one or two identical variants, that were common in European non-Finnish population according to the gnomAD database. In both families with exon4_8dup, no variant was found in the 256 bps long analysed region around the breakpoint.

In addition to variants that were found in close proximity of breakpoints, common *LDLR* variants situated further away from the breakpoints were haplotyped. A list of common variants used for haplotyping can be found in Table 2.

The resulting haplotype is a combination of variants found in close proximity of the breakpoint, and frequent variants in or near *LDLR* exons. The haplotype was obtained in 20 out of 26 families with exon2_6dup, 2 out of 2 families with exon4_8dup, 4 out of 4 families with exon5_10del, 16 out of 28 families with exon9_14del, 8 out of 11 families with exon9_15del, and 5 out of 8 families with exon16_18dup. All the analysed families with a specific rearrangement had the same haplotype associated with the rearrangement. The obtained haplotypes are shown in Table 5.

**Table 5:** Haplotypes associated with specific *LDLR* rearrangements in the Czech population

Rearrangement	Haplotype associated with the rearrangement
Exon2_6dup	exon2_6dup (c.67+3545_940+917dup) – c.940+793_940+795dup – c.1706-55C – c.1725C – c.1773T
Exon4_8dup	c.81C – exon4_8dup (c.314-443_1187-385dup) – c.1773C
Exon5_10del	c.694+285C – c.694+341A – exon5_10del (c.695-67_1586+371del) – c.1706-55C – c.1725C – c.1773T
Exon9_14del	c.81C – c.1186+684C – exon9_14del (c.1186+700_2141-545del) – c.2232G – c.2548-42G
Exon9_15del	c.81C – c.190+56G – c.1187-269_1187-266TG[4] – exon9_15del (c.1187-169_2312-790del) – c.2312-759_2312-754del – c.2548-42G
Exon16_18dup	c.1706-55C – c.1725C – c.1773T – exon16_18dup (c.2312-2067_*1216dup) – c.*1216dup

Haplotypes were obtained in the majority of families with each rearrangement. All analysed families with a specific rearrangement shared the same haplotype as denoted in the table

### Analysis of Alu repeats

To assess the role of Alu repeats in the generation of the rearrangements, the position of repeats within the *LDLR* gene was annotated using RepeatMasker. If the break had occurred in an Alu repeat on both sides of the breakpoint, the sequence identity of the two repeats in which the break occurred was determined using the global alignment tool Emboss Needle.

**Fig. 2:**
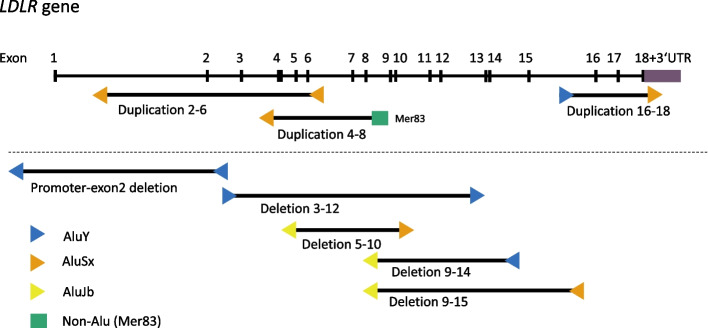
Schematic representation of rearrangements and repetitive elements present at breakpoints. The figure shows the sizes of studied duplications (top part) and deletions (bottom part), along with the character of repeats on each side of the breakpoint. The rearrangements are shown relative to the whole *LDLR* gene, which is shown at the top of the figure. In the *LDLR* schematic, exons are denoted by a vertical line, while the horizontal line represents introns. The orientation of coloured triangles denotes the orientation of Alu repeats at breakpoints, while their colour denotes an Alu repeat subfamily (as determined by RepeatMasker)

The characteristics of repeats present at the breakpoints are shown in Table 6 and Fig. 2.

**Table 6:** Characteristics of repeats present at the breakpoint

Rearrangement	Classification and orientation of repeat 1 ^a^	Classification and orientation of repeat 2 ^a^	Repeats in the same orientation	Chimeric Alu formation ^b^	Homology between the two repeats (%) ^c^	Length of alignment ^d^	Length of microhomology region (bps)
promoter_exon2del	< AluY	< AluY	yes	yes	89	306	42
exon2_6dup	< AluSx	< AluSx	yes	yes	77	305	23
exon3_12del	> AluY	> AluY	yes	yes	91	304	49
exon4_8dup	< AluSx	non-Alu (MER83)	no homology	no	35	99	2
exon5_10del	< AluJb	> AluSx	no	no	35	395	4
exon9_14del	< AluJb	< AluY	yes	yes	87	280	5
exon9_15del	< AluJb	< AluSx	yes	yes	70	142	32
exon16_18dup	> AluY	> AluSx	yes	yes	84	291	0

^a^" > " denotes that the Alu element aligns to the consensus Alu sequence in a 5′➔3' orientation. " < " denotes reverse complementary orientation. Classification of repeats is based on an analysis with RepeatMasker, version open-4.0.9, cross_match mode

^b^Chimeric Alu formation – A chimeric Alu was formed if the break occurred at the same location in both repeats flanking the breakpoint (+-a few bps)

^**c**^Global homology between the two repeats flanking the breakpoint was determined using the EMBOSS Needle tool [28]. (If the polyA tail was longer in one repeat than the other, the extra As were excluded for the sake of calculating the percent homology.)

^d^Length of the alignment that was used to compute percent homology between two repeats

All breakpoints were located inside an Alu element except for one. The duplication of exons 4–8 joined together an Alu element with Mer83, which is an LTR retrotransposon without sequence homology to Alu repeats. In 3 out of 8 rearrangements, the recombination was mediated by a pair of Alu elements belonging to the same subfamily.

In 6 out of 8 rearrangements (2 duplications and 4 deletions), there was high homology (≥ 70%) between the two Alu repeats in which the break occurred. In these cases, the two repeats were in the same orientation and the break occurred in a microhomology region at the same position within the two repeats, creating a new chimeric Alu element. The average percent identity between these 6 pairs of Alu repeats was 83%, ranging from 70 to 91% (Table 6).

In 2 remaining rearrangements (1 duplication and 1 deletion) there was low homology (35%) between repeats surrounding the breakpoint. In the case of exon4_8dup, the recombination occurred between an Alu element and a non-Alu repetitive element Mer83. In the case of exon5_10del, the two Alu repeats flanking the breakpoint were in opposite orientations.

In the case of deletion of exons 9–15, it was hard to compute the percent homology as the breakpoint in intron 8 is inside a partial Alu repeat that has had another Alu repeat inserted inside of it. Taking only the partial Alu surrounding the breakpoint in intron 8 and aligning it with half of the Alu surrounding the breakpoint in intron 15, the homology of these two partial Alus was 70% in a region of 142 bps. The region of (imperfect) homology was shorter than for most other rearrangements reported in this work, but the microhomology (region of perfect homology) was longer.

For all the rearrangements analysed in this publication, the length of the microhomology region present at the breakpoint junction ranged from 0 to 49 bps (Table 6), mean 19.6 bps. The longest microhomology was in the case of exon3_12del (49 bp). The breakpoint of exon16_18dup contained no microhomology.

### NGS CNV analysis

To narrow down the breakpoint position or to confirm a breakpoint determined by Sanger sequencing, CNV analysis of NGS data was performed (Table 7).

**Table 7:** The position of the rearrangement determined by NGS analysis

Rearrangement	CNV position determined by NGS analysis	Genome assembly
promoter_exon2del	NC_000019.10:g.(1064802_11089989)_(11099765_11101419)del	GRCh38/hg38
exon2_6dup	NC_000019.9:g.(11202240_11211067)_(11217915_11221511)dup	GRCh37/hg19
exon3_12del	NC_000019.10:g.(11101200_11113952)_(11118252_11119410)del	GRCh38/hg38
exon4_8dup	NC_000019.10:g.11104775_(11112575_11113852)dup	GRCh38/hg38
exon5_10del	NC_000019.10:g.11106498_11114129del	GRCh38/hg38
exon9_14del	NC_000019.9:g.(11221955_11224202)_(11232040_11233837)del	GRCh37/hg19
exon9_15del	NC_000019.10:g.(11112476_11113952)_(11126051_11128869)del	GRCh38/hg38
exon16_18dup	NC_000019.9:g.(11236220_11237023)_(11241550_11244171)dup	GRCh37/hg19

In cases where the breakpoint was inside a sequenced region, it was sometimes possible to determine the exact location of the breakpoint when viewing the genomic data in IGV. In other cases, only the approximate location of the breakpoint could be determined after analysing the data in QIAGEN CLC Genomics Workbench v.21.0.5

In most cases, NGS CNV analysis did not allow for accurate determination of breakpoint position, due to the method’s inability to reliably sequence repetitive regions, such as Alu repeats, which are plentiful inside the introns of the *LDLR* gene. Thus, the NGS analysis was mostly used to narrow down the breakpoint position within the bounds of a few Alu repeats, sometimes only within a certain intron. In a few cases, the exact location of the breakpoint could be determined by visually examining the data in IGV.

For exon5_10del, it was possible to determine the exact position of the breakpoint using solely NGS analysis. The deleted region determined by NGS was identical to the deleted region determined by Sanger sequencing with the exception that the deletion according to NGS did not include the microhomology region surrounding the breakpoint.

All breakpoints determined by Sanger sequencing in this study fell within the borders determined by the NGS CNV analysis.

## Discussion

### Common ancestry

In the present study, the breakpoints of eight large rearrangements within the *LDLR* gene were characterized in the Czech population. The main goal of this work was to determine whether all Czech families with the same rearrangement carry identical breakpoints inherited from a common ancestor. Sequence analysis of all Czech families carrying exon2_6dup, exon4_8dup, exon5_10del, exon9_14del, exon9_15del, exon16_18dup was performed (with the exception of one proband of exon2_6dup, whose DNA was not available for analysis). (The remaining two rearrangements whose breakpoint was analysed in this publication, namely exon3_12del and promoter_exon2del, were found only in one proband, and thus excluded from this analysis.) For each of these 6 rearrangements, all Czech families with a specific rearrangement shared identical breakpoint positions and sequences.

There are two possible reasons why the breakpoint was identical in all families with the same rearrangement. This could be the result of either a founder mutation in the Czech population or a recurrent rearrangement mediated by the same microhomology region within the same pair of Alu repeats in each family. However, the possibility of a recurrent rearrangement seems less likely. Even though recurrent rearrangements mediated by the same pair of Alu repeats have been reported by Vocke et al. [30], the breakpoint was not the same in all families with the recurrent Alu-mediated rearrangement. Vocke et al. [30] described several recurrent rearrangements occurring in the same pairs of Alu repeats in the VHL gene. Firstly, they described a “hotspot” Alu repeat‐based deletion of exon 3 of the VHL gene. Among 12 families with recombination occurring between the same pair of Alu-repeats, there were 4 distinct (distinguishable) breakpoints. In addition, they described four families with a deletion of exon 2, in which case all four families had a deletion occurring between the same pair of Alu repeats, but different breakpoint positions within these repeats. Three other VHL deletions were each found in two families, with the rearrangement occurring in the same pair of Alu repeats in both families, but with a different breakpoint.

In contrast, among our patients, there were relatively large sets of families with the same breakpoint. Whereas 12 families described in Vocke et al. [30] had four different breakpoints within the same pair of Alu repeats, the current study included as many as 28 families with exon9_14dup and 26 families with exon2_6dup that shared exactly the same breakpoint. If this was a case of a recurrent rearrangement, it seems likely that at least some of these families would have a different breakpoint.

To obtain further evidence for the common origin of these rearrangements in the Czech population, haplotypes associated with the rearrangements in multiple families carrying the same rearrangement were determined, and the results showed that families with the same rearrangement shared the same haplotype.

Based on these findings, it is likely that in all Czech families with duplication of exons 2–6, exons 4–8 and exons 16–18, and the deletion of exons 5–10, 9–14 and 9–15, the rearrangement comes from a common ancestor.

There is also a concerning possibility that a PCR artefact could have arisen during our analysis. This could potentially explain why the resulting breakpoint was the same in all probands with the same rearrangement. However, the fact that all analysed families also had the same haplotype associated with the rearrangement makes this option less likely.

### The role of Alu repeats in the generation of rearrangements

For 7 out of 8 breakpoints, the rearrangement occurred between two Alu repeats. To determine the role of Alu repeats in the generation of these rearrangements, the percent identity of the two Alu repeats was taken into account, as well as the fact whether the break has occurred in the same position in the two repeats or not.

In 6 out of 8 rearrangements (2 duplications and 4 deletions), there was high homology (≥ 70%) between the two Alu repeats in which the break occurred. In these cases, the two repeats were in the same orientation and the break occurred in a microhomology region at the same position within the two repeats, creating a new chimeric Alu element. Based on these observations, Alu elements likely played a role in the generation of these 6 rearrangements. In 3 out of these 6 rearrangements, the recombination was mediated by a pair of Alu elements belonging to the same subfamily.

In the remaining 2 out of 8 rearrangements, there was low homology (35%) between repeats surrounding the breakpoint. The role of Alu repeats in the generation of these rearrangements is uncertain.

Only in one out of 8 rearrangements, the break did not occur in two Alu repeats. In the case of exon4_8dup, one break occurred within an AluSx repeat inside intron 3, and the other one occurred within a short fragment of Mer83 inside intron 8. Mer83 belongs to the class of LTR retrotransposons without sequence homology to Alu repeats. This Mer83 fragment was located between an AluSx repeat and a Tigger4a repetitive element, as identified by RepeatMasker version open-4.0.9. Although the sequence homology of the two repeats in which the break occurred (AluSx and Mer83) was quite low (35%), there was another AluSx element 53 bp upstream of the breakpoint, which was 75.3% identical to the breakpoint Alu in intron 3. There is a possibility that these two Alus could have aligned, contributing to the generation of the rearrangement. The rearrangement could have been initiated by the pairing of two Alu repeats, even if the resulting break occurred elsewhere [22]. Alternatively, the break could have been generated in an Alu-independent manner, and the position of one of the breaks within an Alu element could have been a coincidence due to the high density of Alu repeats within the introns of the *LDLR* gene.

In the case of exon5_10del, the break occurred at the 3’ end of two Alu repeats, which were oriented in opposite directions. The homology of the two repeats in this orientation was only 35%. If one of the repeats is reverse-complemented, the homology is 62%, which is still lower than other Alu pairs in this study. Directly upstream of the breakpoint-Alu in intron 4 is another Alu repeat, which could potentially pair with the breakpoint-Alu in intron 11. However, the homology between these two repeats is only 33%. In conclusion, there does not seem to be any significant homology in the close vicinity of this breakpoint. This deletion probably occurred independently of Alu repeats.

In conclusion, Alu elements likely played a role in the generation of the majority of rearrangements within the *LDLR* gene, but not necessarily all of them.

### Correction of a previous study

The breakpoints of these rearrangements have already been analysed on a smaller number of Czech probands by Goldmann et al. [23]. In the current study, we analysed the breakpoints of these rearrangements with a larger number of probands from the Czech population. We have also made the breakpoints characterised in Goldmann et al. [23] more accurate (Table 3**)**. In 3 cases, the position of the breakpoint differed from that published by Goldmann et al. by several hundred bps (656, 790 and 1044 bps in exon16_18dup, exon3_12del and exon2_6dup, respectively). In one case, the breakpoint position determined in this study differed from the previous study by only a few bps (exon4_8 dup). For exon16_18 dup, the new breakpoint position determined in the current study was also supported by our CNV analysis of NGS data. NGS analysis could narrow down the breakpoint position to a region of 465 bps. This region included the new breakpoint position, but not the previous one. The possible causes of the discrepancies between this study and the previous study are discussed in Additional file 3.

## Strengths and limitations

The strength of this study lies in having access to DNA samples from all the Czech FH patients thanks to the MedPed project. This allowed us to analyse each rearrangement in all the known probands in the Czech population in order to gain new insights into the spread and maintenance of aberrant alleles in the population.

The main limitation of the breakpoint characterization was the inability to explain why the breakpoint of exon2_6dup was different from the previous study [23]. Although other differences in breakpoint placement between these two studies could be explained (see Additional file 3), the source of the discrepancy in the breakpoint placement for exon2_6dup remains elusive. We hypothesised that this discrepancy could have been caused by a PCR artefact but we were unfortunately unable to provide experimental evidence of this artefact.

The haplotype analysis had several limitations. For some rearrangements, the haplotype analysis could not be completed in all families because some probands had no relatives available for analysis. However, a partial haplotype was obtained for families without a full haplotype, and the partial haplotype was consistent with the haplotype determined in other families with the same rearrangement. Table 4 shows a list of variants that were found in *cis* in close proximity of the breakpoint, and those variants were identical in all probands with the same rearrangement.

Another limitation is the fact that the haplotype analysis included a relatively low number of variants, some of which had low allele frequency in the European non-Finnish population, notably c.190+56G>A with a frequency of 0.06341 (Table 2). The conclusions of the study could be strengthened by including more variants in the haplotype analysis.

## Conclusions

The goal of the present study was to analyse the breakpoints of several large rearrangements inside the *LDLR* gene. Sequencing the breakpoints in all Czech probands with each rearrangement has shown that the breakpoint of each rearrangement was the same in all Czech families. In addition, families with a certain rearrangement also had the same haplotype associated with the rearrangement. This result points to the likelihood that these rearrangements originate from a common ancestor, giving us a deeper insight into the genetics of FH in the Czech population.

The finding that Czech patients with a specific *LDLR* rearrangement typically have a specific breakpoint could streamline cascade testing in families of Czech FH patients. Nowadays, the primary method used for detecting large rearrangements in the *LDLR* gene is an NGS analysis or MLPA. However, the use of such cost-intensive methods may not be necessary to confirm the presence of the rearrangement in relatives of patients carrying a known rearrangement. This study opens up an opportunity to use PCR amplification and Sanger sequencing of breakpoints as a time- and cost-effective alternative to NGS or MLPA for confirmation of a specific rearrangement in the Czech population. Primers supplemented in this study can be readily used for this purpose.

Additionally, the breakpoint sequences were analysed for the presence of Alu repeats, revealing that most rearrangements had homologous Alu repeats flanking the breakpoint. In conclusion, Alu repeats could have had a role in the generation of most of these rearrangements.

### Supplementary Information

**Additional file 1.** Primers – Primers used for amplification and Sanger sequencing.

**Additional file 2**. Sequences – Sequences obtained by sequencing the breakpoint region of large rearrangements of the *LDLR* gene.

**Additional file 3**. This file includes a more detailed discussion concerning the discrepancies between the breakpoint positions reported in this study and a previous study [23].

## Data Availability

The datasets supporting the conclusions of this article are included within the article and its additional files, with the exception of raw NGS data, which contain patients’ genomic data with unique variants, for which it may not be possible to guarantee complete anonymity. The sequences surrounding breakpoints that were obtained by Sanger sequencing are included in Additional file 2.

## Abbreviations

APOB: Apolipoprotein B
BLAST: Basic local alignment search tool
CNV: Copy number variation
FH: Familial hypercholesterolemia
IGV: Integrative Genomics Viewer
LDL: Low-density lipoprotein
LDLR: Low-density lipoprotein receptor
MLPA: Multiplex ligation-dependent probe amplification
NAHR: Non-allelic homologous recombination
NGS: Next-generation sequencing
NHEJ: Non-homologous end joining
PCSK9: Proprotein convertase subtilisin/kexin type 9
SINE: Short interspersed nuclear elements
MedPed project: Make Early Diagnosis to Prevent Early Deaths

## References

[1] Beheshti SO, Madsen CM, Varbo A, Nordestgaard BG (2020). Worldwide Prevalence of Familial Hypercholesterolemia. J Am Coll Cardiol.

[2] Hu P, Dharmayat KI, Stevens CAT, Sharabiani MTA, Jones RS, Watts GF, Genest J, Ray KK, Vallejo-Vaz AJ (2020). Prevalence of Familial Hypercholesterolemia Among the General Population and Patients With Atherosclerotic Cardiovascular Disease: A Systematic Review and Meta-Analysis. Circulation.

[3] Yu Y, Chen L, Zhang H, Fu Z, Liu Q, Zhao H, Liu Y, Chen Y. Association Between Familial Hypercholesterolemia and Risk of Cardiovascular Events and Death in Different Cohorts A Meta-Analysis of 1.1 Million Subjects Front Cardiovasc Med. 20229860196 doi 10.3389/fcvm.2022.860196 10.3389/fcvm.2022.860196 PMC9253470 35800161

[4] Slack J (1969). Risks of ischaemic heart-disease in familial hyperlipoproteinaemic states. Lancet.

[5] Stone NJ, Levy RI, Fredrickson DS, Verter J (1974). Coronary artery disease in 116 kindred with familial type II hyperlipoproteinemia. Circulation.

[6] Cuchel M, Raal FJ, Hegele RA, Al-Rasadi K, Arca M, Averna M (2023). 2023 Update on European Atherosclerosis Society Consensus Statement on Homozygous Familial Hypercholesterolaemia: new treatments and clinical guidance. Eur Heart J..

[7] McGowan MP, Hosseini Dehkordi SH, Moriarty PM, Duell PB (2019). Diagnosis and Treatment of Heterozygous Familial Hypercholesterolemia. J Am Heart Assoc.

[8] Tichý L, Fajkusová L, Zapletalová P, Schwarzová L, Vrablík M, Freiberger T (2017). Molecular genetic background of an autosomal dominant hypercholesterolemia in the Czech Republic. Physiol Res.

[9] Bertolini S, Pisciotta L, Rabacchi C, Cefalù AB, Noto D, Fasano T, Signori A, Fresa R, Averna M, Calandra S (2013). Spectrum of mutations and phenotypic expression in patients with autosomal dominant hypercholesterolemia identified in Italy. Atherosclerosis.

[10] van der Graaf A, Avis HJ, Kusters DM, Vissers MN, Hutten BA, Defesche JC, Huijgen R, Fouchier SW, Wijburg FA, Kastelein JJ, Wiegman A (2011). Molecular basis of autosomal dominant hypercholesterolemia: assessment in a large cohort of hypercholesterolemic children. Circulation.

[11] Hobbs HH, Russell DW, Brown MS, Goldstein JL (1990). The LDL receptor locus in familial hypercholesterolemia: mutational analysis of a membrane protein. Annu Rev Genet.

[12] Amsellem S, Briffaut D, Carrié A, Rabès JP, Girardet JP, Fredenrich A, Moulin P, Krempf M, Reznik Y, Vialettes B, de Gennes JL, Brukert E, Benlian P (2002). Intronic mutations outside of Alu-repeat-rich domains of the LDL receptor gene are a cause of familial hypercholesterolemia. Hum Genet.

[13] Lander ES, Linton LM,  Birren  B, Nusbaum  C, Zody  MC, Baldwin  J (2001). International Human Genome Sequencing Consortium. Initial sequencing and analysis of the human genome. Nature.

[14] Houck CM, Rinehart FP, Schmid CW (1979). A ubiquitous family of repeated DNA sequences in the human genome. J Mol Biol.

[15] Ullu E, Tschudi C (1984). Alu sequences are processed 7SL RNA genes. Nature.

[16] Batzer MA, Deininger PL (2002). Alu repeats and human genomic diversity. Nat Rev Genet.

[17] Lehrman MA, Schneider WJ, Südhof TC, Brown MS, Goldstein JL, Russell DW (1985). Mutation in LDL receptor: Alu-Alu recombination deletes exons encoding transmembrane and cytoplasmic domains. Science.

[18] Gu W, Zhang F, Lupski JR (2008). Mechanisms for human genomic rearrangements. Pathogenetics.

[19] de Smith AJ, Walters RG, Coin LJ, Steinfeld I, Yakhini Z, Sladek R, Froguel P, Blakemore AI (2008). Small deletion variants have stable breakpoints commonly associated with alu elements. PLoS ONE.

[20] Boone PM, Yuan B, Campbell IM, Scull JC, Withers MA, Baggett BC, Beck CR, Shaw CJ, Stankiewicz P, Moretti P, Goodwin WE, Hein N, Fink JK, Seong MW, Seo SH, Park SS, Karbassi ID, Batish SD, Ordóñez-Ugalde A, Quintáns B, Sobrido MJ, Stemmler S, Lupski JR (2014). The Alu-rich genomic architecture of SPAST predisposes to diverse and functionally distinct disease-associated CNV alleles. Am J Hum Genet.

[21] Gu S, Yuan B, Campbell IM, Beck CR, Carvalho CM, Nagamani SC, Erez A, Patel A, Bacino CA, Shaw CA, Stankiewicz P, Cheung SW, Bi W, Lupski JR. Alu-mediated diverse and complex pathogenic copy-number variants within human chromosome 17 at p13.3. Hum Mol Genet. 2015;24(14):4061–77. doi: 10.1093/hmg/ddv146 10.1093/hmg/ddv146 PMC4476451 25908615

[22] Morales ME, White TB, Streva VA, DeFreece CB, Hedges DJ, Deininger PL (2015). The contribution of alu elements to mutagenic DNA double-strand break repair. PLoS Genet.

[23] Goldmann R, Tichý L, Freiberger T, Zapletalová P, Letocha O, Soska V, Fajkus J, Fajkusová L (2010). Genomic characterization of large rearrangements of the LDLR gene in Czech patients with familial hypercholesterolemia. BMC Med Genet.

[24] Vrablík M, Vaclová M, Tichý L, Soška V, Bláha V, Fajkusová L, Češka R, Šatný M, Freiberger T (2017). Familial hypercholesterolemia in the Czech Republic: more than 17 years of systematic screening within the MedPed project. Physiol Res.

[25] Tichý L, Freiberger T, Zapletalová P, Soška V, Ravčuková B, Fajkusová L (2012). The molecular basis of familial hypercholesterolemia in the Czech Republic: spectrum of LDLR mutations and genotype-phenotype correlations. Atherosclerosis.

[26] Altschul SF, Gish W, Miller W, Myers EW, Lipman DJ (1990). Basic local alignment search tool. J Mol Biol.

[27] Smit AFA, Hubley R, Green P. RepeatMasker. https://www.repeatmasker.org/cgi-bin/WEBRepeatMasker [Accessed 25 October2020]

[28] Madeira F, Park YM, Lee J, Buso N, Gur T, Madhusoodanan N, Basutkar P, Tivey ARN, Potter SC, Finn RD, Lopez R (2019). The EMBL-EBI search and sequence analysis tools APIs in 2019. Nucleic Acids Res.

[29] Robinson JT, Thorvaldsdóttir H, Winckler W, Guttman M, Lander ES, Getz G, Mesirov JP (2011). Integrative genomics viewer. Nat Biotechnol.

[30] Vocke CD, Ricketts CJ, Schmidt LS, Ball MW, Middelton LA, Zbar B, Linehan WM (2021). Comprehensive characterization of Alu-mediated breakpoints in germline VHL gene deletions and rearrangements in patients from 71 VHL families. Hum Mutat.

[31] Konečná K, Tichý L, Freiberger T. Common rearrangements of the LDLR gene in the Czech population likely arise from one mutational event. Poster session presented at European Human Genetics Conference; June 11–14 2022; Vienna, Austria. Abstracts from the 55th European Society of Human Genetics (ESHG) Conference: Hybrid Posters. Eur J Hum Genet. 2023;31:412–3. 10.1038/s41431-023-01338-4.

