# Supplementary material for: LDLR gene rearrangements in Czech FH patients likely arise from one mutational event
Source: Lipids Health Dis. 2024 Feb 2;23:36. doi: 10.1186/s12944-024-02013-3 (PMC10835926; doi:10.1186/s12944-024-02013-3)
Supplement: Supplementary file 1 — Additional file 1. Primers – Primers used for amplification and Sanger sequencing. [file 12944_2024_2013_MOESM1_ESM.docx]

**Supplementary Table S1: Primers used for amplification and sequencing of the breakpoints**

| Rearrangement | Outer primer pair ^1^ | Inner primer pair | PCR product size (inner primer pair) (bps) ^4^ | Length of the sequenced region (bps) ^5^ |
| --- | --- | --- | --- | --- |
| promoter_exon2del | TGACACACACTGAGCCTACG | GCAGCTCCTAGAACTTGCCT | 361 | 229 |
|  | TATGGGTCAAAACGGAGGGC | ATAGGCCGGGCATGGTG ^3^ |  |  |
| exon2_6dup | - | CCTTAGATGCCTGCTTCTGTCT | 401 | 401 |
|  | - | ATCCCCAGATTTTCTCACGCT ^3^ |  |  |
| exon3_12del | - | AAAAAGTGGGATTAGGTCAGGCA ^3^ | 642 | 215 |
|  | - | TGGCCCACAACTAGTTTTTAATTG ^3^ |  |  |
| exon4_8dup | - | GGAGTGACTTCAAGGGGTTAAAG ^2^ | 1117 | 256 |
|  | - | TTCTCTAAAATGCTTGGGACCA ^2^ ^3^ |  |  |
| exon5_10del | - | GCAGTGATTTAGGTGCCGAAG | 996 | 759 |
|  | - | CTCACGTGCTTAGGTAGCAGA ^3^ |  |  |
| exon9_14del | ACAAGTGCCAGTGTGAGGAA | GGAGTGACTTCAAGGGGTTAAAG ^2^ | 532 | 277 |
|  | GGCAACGAATGTGCCTTGAT | AGGTGGCTCAGGCTGGGC ^2^ ^3^ |  |  |
| exon9_15del | - | CACGTGATCGTCCCGCCTA ^2^ | 831 | 446 |
|  | - | AAATTCTTGTCAACCTACTTGTGC ^2^ ^3^ |  |  |
| exon16_18dup | - | ACTTCAAAGCCGTGATCGTG ^3^ | 579 | 579 |
|  | - | ACGAATGACATCCTGACCCAC ^3^ |  |  |

^1^ Outer primer pair is denoted in cases of nested PCR; ^2^ denotes primers that were identical to those used by Goldmann et al., 2010; ^3^ denotes the primer used for sequencing; ^4^ Exact size of the PCR product was determined based on the sequence obtained by Sanger sequencing. (E.g. If the sequence contained a 3-bps duplication compared to the reference sequence (as was the case for exon2_6dup), the duplication was included in the product size.) The theoretical product size corresponded to the size observed on an agarose gel; ^5^ Length of the region where sequence could be obtained with confidence. Sequencing was often hindered by the presence of long poly-A/poly-T tracks, which are a typical feature of Alu elements, thus it was usually not possible to sequence the whole product.

**Supplementary Table S2: Primers used for the amplification of *LDLR* exons for haplotyping of common sequence variants**

| Exon | Primer F | Primer R |
| --- | --- | --- |
| 2 | TTGGCAGGAAATAGACACAGGA | ACCAGAAATTCAAGACCAGCCT |
| 12 | GGTGCTTTTCTGCTAGGTCCC | TCACAACCAGTTTTCTGCGTTC |
| 15 | GGCCTCCCAAGGTCATTTGA | CTCCGTGACCAAAATGTTCGTG |
| 18 | GTACTCACCGTCTCCCTCTGGC | ACAAAGCTCTGGCAGGCAATG |
