# Supplementary material for: LDLR gene rearrangements in Czech FH patients likely arise from one mutational event
Source: Lipids Health Dis. 2024 Feb 2;23:36. doi: 10.1186/s12944-024-02013-3 (PMC10835926; doi:10.1186/s12944-024-02013-3)
Supplement: Supplementary file 2 — Additional file 2. Sequences – Sequences obtained by sequencing the breakpoint region of large rearrangements of the LDLR gene. [file 12944_2024_2013_MOESM2_ESM.docx]

**Supplementary figure 1: Sequences obtained by sequencing the breakpoint region of large rearrangements of the *LDLR* gene**

**> LDLR**|**Promoter_exon2del breakpoint; start: LDLR promoter, end: intron 2**

TTTTTTTTTTTTTTGAGACGGAGTCTCACTCTGTCGCCCAGGCTGGAGTACAGTGGCGCAATCTCGTCTCACTGCAAACTCCACCTCCCGGGTTCACGCCATTCTCCTGCCTCAGCCTCCCGAGTAGCTGGGACTACAGGCACCCTCCACCACATCCGGCTAATTTTTTGTATCTTTAGTAGAGATGGGGTTTCACCATGTTAGCCGGGATGGTCTCGATCTCCTGACC

**>LDLR**|**Exon2_6dup breakpoint; start: intron 6, end: intron 1**

CCTTAGATGCCTGCTTCTGTCTTGAGGTTTGTTGTTGTTGTTATTTCGAAACAGAGTCTTGCTCTGTCGCTCAGGCTGGAGTGCAGTGGCATGATCTTGGCTCACCACAACCTCCGGCTCCCAGGTTCAAGCGATTCTTCTGCCTCAGCCTCCTGAGTAGCTGGAACTACAGGCGCCCGCCACCACACCCAGCTAATTTTTGTATTTTTCAGTAGAGATGGGATTTCACCATATTGGTCAAGCTGGCCTCGAACTCCTGACCTCGTGATCCACCCGCCTCAGTTTCCCAAAGTGCTGGGAGTACAGGCGTGAGCCGCCGTGCCCGGCCTTTTTGTGTTTTTGTGTTTTTGAGAGGAGCTCATTGCTTTTTAGGCTTCCCTAGCGTGAGAAAATCTGGGGAT

**>LDLR**|**Exon3_12del breakpoint; start: intron 2, end: intron 12**

TCCTGGCTAACACGGTGAAACCCCGTCTCTACTAAAAAAATACAAAAAATTAGCCAGGCGTGGTGGCGGGTGCCTGTAGTCCCAGCTACTCAGGAGGCTGAGGCAGGAGAATGGCGTGAACCCGGGAGGCGGAGCTTGCAGTGAGCCAAGATCGCGCCACTGCACTCCAGCCTGGGTGACAGAGTTGAGACTCCGTCTCAAAAAAAAAAAAAAAA

**>LDLR**|**Exon4_8dup breakpoint; start: intron 8, end: intron 3**

TTTTTTTTTTTTTTTTTGAGACAGAGTCTCATTCTGTCACCCAGGCTGGAGTGCGGTGGTGCGATCTTGGCTTACTGAAACCTCCACCTCCCAGGTTCCAGCAATTCTCCTGCCTCACCCTTCTGAGTAGCTGGGATTACAGGTTCCGGCTACCAAACCTGGCTAGTTTTTGTATGTTTAGTAGAGTCAGTGCTCACCCACAACATTAAGGATATTCCAAATTTGAAACATTCCAAAATCAGAAAAATATTCCAAC

**>LDLR**|**Exon5_10del breakpoint; start: intron 4, end: intron 10**

CTGTTAGCTTATTGGGAAATCCCTGTTTGGAAGGTGCTGGTTGTTTTTTGTTGTTTGTTGTTTTTGTTTTTGTTTTTATTTTGAGACGGAGTCTCGCTCTGTCGCCAGGGTGGAGTGCAGTGGCGCGATCAGCTCACTGCAACCTCCGCTTCCTGGGTTCAAGCCATTCTCCTGCCTCAGCCTCCCAAGTAGCGCGGATTACAGGCATGTGCCACCACCTCCGGCTATTTTTTTTTCTATTTAGTAGAGATGGGGTTTCACCATGTTAGTCAGGCTGGTCATGAACTCTTGACCTCAGGTGATCCACCCGCCTCGGCCTCCCAAAGTGCTGGGATTACAGGCGTGCACTGCTGCACCCAGCCTTTTTTTGTTTTTTTGAGACAGGGTCTTGCTGTCACCCAGGTTGAAGTAAGGTGGCACGATTATGGCTCACTGCGGCCTTGATCTCCTTGGCTCAAGCGATCCTCTCACTTCAGCCTCTCAAGCAGTTGGAACCACAGGCTGTACCACCAAGCCTGGCCAATTTTTTTGTACAGACACAGGCTGGTCTTGAACTCCTGGGCTCAAGCAATCCTCCTGCCTTGGCCTCCCAAAGTGCTGGGATTCCAGGCATGAGCCGCTGCACCCGGCAAAAACTGGTTAGTGGCTAGACAACAGGATGGTATCTTCCAAGCCCATGGCTGACTCAGCAGCTCCTGGGTCAAGACACTGTGACCTGTGTCCCCTGGCAGGAAGCATCGCCCCTGCCACCTGCCCGGT

**>LDLR**|**Exon9_14del breakpoint; start: intron 8, end: intron 14**

TTTTTTTTTTTCGAGACGGAGTCTCGCTCTGTTGCCCAGGCTGGAGTGCAGTGGCAGGATCTCGGCTCACTGCAAGCTCCGCCTCCCGGGTTCACGCCATTCTCCTGCCTCAGCCTCCCCAGTAGCTGGGACTACAGGAGCCCGCCACTGCACCAGGCCAATTTTTTTGTATTTTTAGTAGAGACGGGGTTTCACTGTGTTAGCCAGGCTGGTCTCGATCTCCTAACCTCAGGCGATTCACCTGCCTCGGCCTCCCAAAGTGCTGGGATTAAAGGTA

**>LDLR**|**Exon9_15del breakpoint; start: intron 8, end: intron 15**

TTTTTTTTTTTTTTTTTTTTGTGTGTGGCAATAAGGTCTCATTGTCTTGCCCAGGCTAGCCTTATGCTCCTAGCCTCAAGTGATCCTCCTCCCTCAGCCTCCCAAAGTGCTGGGATTACAGGTTTGAGCCACTGGGCCTGGCCTTTTTTTTTTGAGAGGGAGTCTCACTCTGTTGCCCAGGCTGGAGTGCAATGGCGCGATCTTGACTCACTGCAACTCCATTTCCCGGGTTCAAGTGATTCTCCTCCCTCAGCCTCCCAAGTAGCTGGGATTACAGGTGCATGCCACCACGGCCAGCTAATTTTGTATTTTTAGTAGAGACAGGGTTTCACTATGTTGATCATGCTGGTCTCAAACTCCTGACCTTAGGTGATCTGCCCGCCTTAGCCTCCCAAAGTGTTGGGATTACAGGTGTGAGCCACCGCGCCCAGACCAAAATATGCTCA

The red box denotes the region of microhomology containing the breakpoint. This sequence is present in both introns in which the break occurred. To the left of the breakpoint region is a sequence homologous to the reference sequence of the first intron denoted above the sequence, while to the right of the breakpoint is a sequence homologous to the second intron denoted above the sequence. Blue, underlined boxes denote sequence variants that differ from the reference sequence NG_009060.1(NM_000527.4) that were found in sequenced samples. All analysed samples shared the same variants.

**Supplementary figure 2: Sequence obtained by sequencing the breakpoint region of exon16_18dup**

**>LDLR**|**Exon16_18dup start: exon 18 (3’UTR), end: intron 15**

ACTTCAAAGCCGTGATCGTGAATATCGAGAACTGCCATTGTCGTCTTTATGTCCGCCCACCTAGTGCTTCCACTTCTATGCAAATGCCTCCAAGCCATTCACTTCCCCAATCTTGTCGTTGATGGGTATGTGTTTAAAACATGCACGGTGAGGCCGGGCGCAGTGGCTCACGCCTGTAATCCCAGCACTTTGGGAGGCCGAGGCGGGTGGATCATGAGGTCAGGAGATCGAGACCATCCTGGCTAACAAGGTGAAACCCCGTCTCTACTAAAAATACAAAAATTAGCCAGGCGTGGTGGCAGGTGCCTGTAATCCCAGCTACTCGGGAGGCTGAAGCACGAGAATCGCTTGAATCCAGGAGGCGGAGGTTGCAGTGAGCTGAGATTGCGCCATTGCACTCCAGCCTGGAGGACAAGAGTGAAACTCCATTCCCCTCTGCAAAGAAAAGGAATATTATCAGATTCCTAAGCTTTTTGGCTCCCCCTTTAGTTTGGGGGCTGGGGTGGTGAGTGTCTGACCTGGCCTCACTGTCCTCCCTGGATGTGATGAGACCCAGGTGTGGGTCAGGATGTCATTCGT

In the case of exon16_18dup, there was no microhomology at the breakpoint site, thus the exact position of the break *could* be determined. In position -1 relative to the breakpoint, there is a duplication of one adenine (underlined blue box), which could be either a microduplication that arose during the generation of the rearrangement, or the common variant c.*1216dupA, which is found with an allele frequency of 68% in the European non-Finnish population according to gnomAD database v2.1.1. The sequence left of the breakpoint corresponds to the reference sequence of exon 18 (3’UTR), while the sequence right of the breakpoint corresponds to the reference sequence of intron 15 (reference sequence NG_009060.1(NM_000527.4)).
