# Supplementary material for: LDLR gene rearrangements in Czech FH patients likely arise from one mutational event
Source: Lipids Health Dis. 2024 Feb 2;23:36. doi: 10.1186/s12944-024-02013-3 (PMC10835926; doi:10.1186/s12944-024-02013-3)
Supplement: Supplementary file 3 — Additional file 3. This file includes a more detailed discussion concerning the discrepancies between the breakpoint positions reported in this study and a previous study [23]. [file 12944_2024_2013_MOESM3_ESM.docx]

# Correction of a previous study

The breakpoints of these rearrangements had already been analysed on a smaller number of Czech probands in a previous study by Goldmann et al. [1]. In the current study, we analysed the breakpoints of these rearrangements with a considerably larger number of probands from the Czech population. Surprisingly, some of the breakpoint sequences obtained in this study were different from the previous ones, even though the current study also included the samples that were used in the previous study.

The original goal of the current study was to determine the breakpoint of all probands with exon2_6dup in the Czech population to determine if all probands carry the same breakpoint inherited from a common ancestor. We used the previous study as a reference. We were able to analyse 26 out of 27 known probands with exon2_6dup, and we obtained the same breakpoint sequence in all probands, which was however different from the breakpoint sequence published by Goldmann et al. Considering that we used the same samples, it was not possible for the breakpoints to be different.

We concluded that the breakpoint of exon2_6dup published by Goldmann et al. needed to be corrected based on the following observations. When amplifying the breakpoint region using the primers published by Goldmann et al., the size of the PCR product on an agarose gel was about 1,000 bps larger than expected based on the breakpoint location determined by Goldmann et al. We attempted to sequence the breakpoint site delineated by Goldmann et al., but a normal sequence of intron 1 or 6 continued past the putative breakpoint. We designed a new pair of primers which annealed to the part of the gene that was not supposed to be a part of the duplication based on Goldmann’s breakpoint (Additional File 1). If Goldmann’s breakpoint was correct, the annealing region of the new primers would not be included in the duplicated region so primers would not be able to align in close proximity and yield a PCR product. However, we obtained a product with the new primers, and sequencing the product showed a new breakpoint sequence.

Based on this finding, we decided to verify the breakpoint sequence in all rearrangements published by Goldmann et al. [1]. The results showed that the breakpoint sequence and position determined in this study were the same as that of Goldmann et al. in 4 out of 8 rearrangements. In 3 cases, the position of the breakpoint differed by several hundred bps (656, 790 and 1,044 bps in exon16_18dup, exon3_12del and exon2_6dup, respectively), while in the case of exon4_8dup, there was only a difference in the theoretical deduction of breakpoint position from the same sequence, and we determined the breakpoint position to be just a few bps different.

For exon16_18dup, raw data from Goldmann et al. showed the same sequence that we obtained during reanalysis, although table 2 and figure 2 in Goldmann et al. show a different breakpoint. In this case, the difference was apparently caused by differences in the theoretical deduction of the breakpoint position from the same raw sequence. The breakpoint position determined by this study was also supported by our CNV analysis of NGS data. NGS analysis could narrow down the breakpoint position to a region of 465 bps. This region included the new breakpoint position, but not the previous one.

For exon2_6dup, we were not able to clearly determine how Goldmann et al. could have obtained a different breakpoint. Because this breakpoint also appeared in their raw sequencing data, we suspected it might have been the result of a PCR artefact, where a shortened product could have arisen during PCR, perhaps as a result of the high Alu-repeat density in the amplified region. Kurahashi et al. [2] described a case of an Alu-mediated deletion that arose during PCR and concealed the true breakpoint. They provided the explanation that the PCR polymerase could potentially get stuck at a hard-to-amplify region. In the next cycle, this incomplete product could serve as a primer and misalign to another location complementary to its 3’ end, potentially another Alu repeat. Thus, Alu-mediated deletion artefacts might be a possible cause of the discrepancy between the breakpoint reported by Goldmann et al. and the newly established breakpoint.

In the case of exon3_12del, we concluded that the breakpoint published by Goldmann et al. could not have been correct based on the primers reported in the previous study. Goldmann et al. used an R primer (TGGCTCACTGCAAGCTCCG) that was not specific to the deletion allele because it had 100% complementarity to two sites within the LDLR gene – one in intron 12 (the intended target), and one in intron 2 in such a position that it could have potentially created a product with the F primer (which was also targeting intron 2), amplifying the normal intron 2 (unintended target). This R primer annealed to the microhomology region of the breakpoint as determined by our analysis, meaning it would theoretically create an identical product from the deletion allele and WT allele. The breakpoint sequence of exon3_12del published in Goldmann et al., 2010 appears to be a sequence of intron 2 (putative product of F and R primers both annealing to intron 2), from which 790 bps has been deleted. The mechanism behind the generation of this deletion could have possibly been similar to the putative PCR artefact that hypothetically arose in their analysis of exon2_6dup. The size of the long-range product for exon3_12del stated in Goldmann’s table 1 does not correspond to the breakpoint location they published in table 2. If this breakpoint location was correct, the size of the product with this pair of primers should have been 2.5 kbps, not 3.5 kbps as stated in the table. The 3.5 kbps product size corresponds to the breakpoint location determined in the current study.

In conclusion, a combination of various causes produced inaccurate results in the previous study by Goldmann et al., which we have uncovered and corrected through careful extended analysis.

# References

1. Goldmann R, Tichý L, Freiberger T, Zapletalová P, Letocha O, Soska V, Fajkus J, Fajkusová L. Genomic characterization of large rearrangements of the LDLR gene in Czech patients with familial hypercholesterolemia. BMC Med Genet. 2010;11:115. doi: 10.1186/1471-2350-11-115
2. Kurahashi H, Shaikh TH, Emanuel BS. Alu-mediated PCR artifacts and the constitutional t(11;22) breakpoint. Hum Mol Genet. 2000;9(18):2727-32. doi: 10.1093/hmg/9.18.2727
